# Human endothelial cells size‐select their secretory granules for exocytosis to modulate their functional output

**DOI:** 10.1111/jth.14634

**Published:** 2019-10-02

**Authors:** Jessica J. McCormack, Kimberly J. Harrison‐Lavoie, Daniel F. Cutler

**Affiliations:** ^1^ MRC Laboratory of Molecular Cell Biology University College London London UK

**Keywords:** endothelial cells, exocytosis, organelle size, von Willebrand factor, Weibel‐Palade bodies

## Abstract

**Background:**

The secretory granules of endothelial cells, Weibel‐Palade bodies, are released in response to numerous extracellular signals. Their cargo is critical to many vascular functions including hemostasis and inflammation. This presents a fundamental problem: how can these cells initiate tailor‐made responses from the release of a single type of organelle, each with similar cargo? Each cell contains Weibel‐Palade bodies in a wide range of sizes, and we have shown that experimentally shortening these organelles disproportionately reduces their ability to initiate hemostasis in vitro, leaving leukocyte recruitment unaffected. Could the production of this range of sizes underpin differential responses?

**Objectives:**

To determine whether different agonists drive the exocytosis of different sizes of Weibel‐Palade bodies.

**Methods:**

We used a high‐throughput automated unbiased imaging workflow to analyze the sizes of Weibel‐Palade bodies within human umbilical vein endothelial cells (HUVECs) before and after agonist activation to determine changes in organelle size distributions.

**Results:**

We found that a subset of agonists differentially evoke the release of the longest, most pro‐hemostatic organelles. Inhibiting the release of these longest organelles by just 15% gives a fall of 60% in an assay of secreted von Willebrand factor (vWF) function.

**Conclusions:**

The size‐selection of granules for exocytosis represents a novel layer of control, allowing endothelial cells to provide diverse responses to different signals via the release of a single type of organelle.

**Essentials**

- The secretory granules of endothelial cells release cargo to support multiple processes.
- Each cell contains granules in a range of sizes, correlating with hemostatic activity.
- Some agonists recruit an actin ring to induce exocytosis of the longest granules.
- Such exocytic selectivity provides a novel layer of control over endothelial output.

## 1 INTRODUCTION

The release of secretory granules controls physiologically critical processes; thus, regulation of secretory responses is essential. However, how this is managed in cells in which exocytosis provides diverse responses is not fully understood. Processes regulated via release of endothelial secretory granules, Weibel‐Palade bodies (WPBs), are numerous and diverse^1^, ^2^, ^3^: The endothelium responds to about 30 agonists that communicate the need to repair damage by initiating hemostasis, respond to invading pathogens by eliciting an inflammatory response, as well as regulating angiogenesis and vascular tonicity.^2^, ^4^ Such a range of functions indicates a likely need to provide tailored responses to different agonists, but how is this achieved? One layer of control is at exocytosis; the magnitude of response can be regulated via the number of exocytic events, the mode of fusion—eg, kiss and run or full fusion of single or multiple granules—and in some systems by differential packaging (and exocytosis) of secretory cargo.^5^ However, these forms of regulation may still be insufficient in more complicated scenarios and may not be universally available. In the case of the endothelium, modulating exocytic magnitude and mode of fusion cannot easily generate appropriate outputs to support the demonstrably wide range of functions these cells are capable of controlling.

A feature of WPBs is that their length (ie, size) is non‐normally distributed and ranges ten‐fold in steps from 0.5 to more than 5 μm. This is determined by the extent of linear copackaging of presized, half‐micron‐long quanta of their major cargo protein, von Willebrand factor (vWF), which drives the formation of these organelles.^6^ WPB size is set at the trans‐Golgi and endothelial cells have considerable influence over this feature; their size is controlled by the size of Golgi cisternae that determines vWF quantal size, by the level of expression of vWF that controls the number of quanta and thus the probability of quanta co‐packaging, and by the degree of linkage of the Golgi ribbon; unlinking the Golgi into separate mini‐stacks precludes copackaging, giving shortened, “mini” WPBs .^6^ Finally, we recently reported that the number of quanta at the trans‐Golgi are also controlled by environmental cues. AMP‐activated protein kinase (AMPK) is activated in response to multiple environmental signals and when active can regulate flux through the secretory pathway, thus linking physiological signals to WPB size.^7^

Initiating hemostasis depends on plasma‐membrane‐anchored vWF strings acting as mechanosensitive binding platforms for the recruitment of platelets and plasma vWF.^8^, ^9^ Importantly, the effect of changes in length of WPBs on the function of vWF is amplified as compared to other cargoes.^6^, ^8^ Cells with fewer long WPBs exhibit disproportionately diminished length and number of VWF strings. Interestingly, the substantial fall in prohemostatic capacity does not simply reflect a reduction in total release of vWF (this is not changed, or can even be increased) and is rather a failure in string assembly.

We, and others, have previously found that an actin ring is recruited at a subset of exocytic events where it is required for the efficient release of vWF.^10^, ^11^, ^12^ Acute inhibition of actin or protein kinase C (PKC) can block ring‐dependent release.^10^, ^13^ Ring recruitment specifically aids the release of vWF, while having little effect on release of smaller molecules stored with WPBs.^13^ Subsequently we found that agonists differentially recruit the ring.^13^ Potentially, actin ring recruitment might act as a means of fine‐tuning the endothelial response, to bias it toward increased prohemostatic functioning.

We therefore addressed the hypothesis that endothelial cells exploit the large range of WPB lengths by size‐selecting those that undergo exocytosis, to increase control over output. Our experiments show that some agonists can preferentially evoke exocytosis of longer WPBs by recruitment of an actin ring. This introduces a novel layer of control over the functioning of endothelial secretory output.

## 2 METHODS

### 2.1 Cell culture and nucleofection

Human umbilical vein endothelial cells (HUVECs) were derived from pooled donors and purchased from Lonza or Promocell. HUVECs were cultured in M199 (Gibco), with 20% fetal calf serum (Biosera), 10 U/mL heparin, and 30 μg/mL endothelial cell growth supplement (both from Sigma). siRNA against Luciferase (5′‐CGUACGCGGAAUACUUCGA[dT][dT]‐3′) and vWF (5′‐ GGGCUCGAGUGUACCAAAA[dT][dT]‐3′) were purchased from Eurofins. siRNA (25pmol) was delivered via nucleofection using the program U‐001 (Lonza). Cells were typically assayed 48 hours after nucleofection.

### 2.2 Antibodies and reagents

Rabbit anti‐vWF (cat. No. A0082) and rabbit anti‐vWF‐HRP, which stain both processed and unprocessed forms of vWF, were from DAKO (termed “total vWF”), two anti‐vWF antibodies against a neo‐epitope exposed at the carboxy terminus of the propeptide upon furin cleavage^14^ to visualize processed vWF (termed “pro‐vWF”), were used in this study. These "pro‐vWF" antibodies allowed visualization of only that vWF packaged into WPBs (thus excluding background staining of ER‐localized vWF). the first was a kind gift from Dr T Carter (St. George's University London), the second was produced by Eurogentec. Hoechst 33342 was from Life Technologies. Cytochalasin E (CCE) was purchased from Sigma, GÖ6976 was from TOCRIS. Histamine was from Enzo Life Sciences (cat. No. ALX‐550‐132); Phorbol 12‐Myristate 13‐Acetate (PMA), adrenaline, and 3‐isobutyl‐1‐metylxanthine (IBMX) were from Sigma; thrombin was from Calbiochem.

### 2.3 WPB release assay

Human umbilical vein endothelial cells were grown in 96 well plates (Nunc) for 2 days. Confluent monolayers were washed in pre warmed release medium (M199 with 0.2% BSA and 10 mmol/L HEPES), and where necessary incubated with or without CCE (0.5 μmol/L) or PKC inhibitor GӦ6976 (1 μm) for 10 minutes. Cells were stimulated with histamine (100 μmol/L), PMA (100 ng/mL), thrombin (1 U/μL), adrenaline (100 μmol/L), or IBMX (10 μmol/L) either alone or in combination for 10‐30 minutes before fixation in 4% paraformaldehyde (PFA). All incubations prior to fixation were carried out at 37°C. Cells were permeabilised in 0.2% Triton X‐100, blocked in 5% bovine serum albumin for 10 minutes each. Staining for pro‐vWF was done using anti‐vWF antibody followed by anti‐rabbit antibody conjugated to Alexa Fluor 488 nm and the nucleus with Hoechst 33342.

### 2.4 Exocytic site labelling assay

Exocytic sites were labeled as described^13^ using a modified method from Knop and Gerke.^15^ Briefly, HUVECs were grown in 96 well plates and when confluent, incubated overnight in either dimethyl sulfoxide (DMSO), acetate pH 6.4 medium, or nocodazole (1 μg/mL). Cells were prepared as for WPB release assay before incubation in the presence of anti‐vWF (DAKO) in the presence or absence of agonist for 10 minutes. Cells were fixed as described above. vWF was visualized by incubation with anti‐rabbit antibody conjugated to Alexa Fluor 488 nm.

### 2.5 High throughput image acquisition and segmentation

The Opera high‐content screening confocal microscope (PerkinElmer) was used to acquire images from cells grown on 96 well plates stored in phosphate‐buffered saline (PBS) using a 40× air objective (NA 0.6). Imaging was carried out at room temperature. Nine fields of view were acquired per well and at least eight wells per condition were used. Images were converted from flex to 16‐bit tiff and analyzed using a custom‐designed program using version 2.7 of the Python programming language with the scikit image library. For segmentation of exocytic sites image noise was reduced with a Gaussian blurring with a sigma value not impacting the image resolution. A binary mask was created using a threshold value obtained from Moment‐preserving thresholding.^16^ Adjacent exit sites were split using the marker‐based watershed flooding algorithm. For segmentation of WPBs a local adaptive threshold was applied.^6^ Segmented objects beneath the resolution limit of the optical system were removed and morphometric measurements taken. The automated segmentation was validated by comparison to a gold standard set of nine images annotated manually. Data analyses were performed in RStudio version 1.1.423.

### 2.6 Secretion assay and ELISA

Human umbilical vein endothelial cells manipulated to alter the population of small WPBs by being grown overnight in DMSO, acetate pH 6.4 medium or nocodazole (1 μg/mL)^6^ or by nucleofection of siRNAs against either Luciferase or vWF were washed in release medium as for WPB release assays. Cells were then stimulated, in the presence or absence of CCE (0.5 μmol/L), with either histamine, histamine in combination with adrenaline and IBMX, or PMA and media recovered. vWF levels in releasate and lysate was determined by ELISA. 96 well plates (Thermo Fisher Scientific) were coated overnight with anti‐vWF (total vWF, DAKO) diluted in PBS. Plates were blocked with 1×TEB (1% Triton x‐100, 0.2% fish skin gelatin, 1 mmol/L EDTA in PBS) for one hour at room temperature. Samples (loaded in duplicate) were incubated for 1 hour at room temperature in 1×TEB. Following three washes in 1×TEB plates were incubated with anti‐vWF antibody conjugated to horseradish peroxidase (HRP); HRP activity was visualized using *o*‐phenylenediamine and read using a Molecular devices plate reader (Versa Max) over 30 minutes at 425 nm.

### 2.7 vWF plasma recruitment assay

Human umbilical vein endothelial cells grown to confluence onto μ‐slides (Ibidi) with a 5 mm‐wide channel were maintained at 37°C and slides attached to a syringe pump (Harvard Apparatus) to draw fluid over cells at a constant wall shear stress of 0.25 MPa (2.5 dynes/cm^2^). Cells were rinsed with Hanks balanced salt solution (HBSS, Life Technologies containing Ca^2+^, Mg^2+^ and 0.2% BSA) for 2 minutes before being stimulated with histamine in combination with adrenaline and IBMX in the presence or absence of CCE (0.25 μm) for five minutes in HBSS followed by normal human pooled plasma for a further five minutes. Cells were fixed with 4% PFA under decreasing rates of flow and subsequently stained for surface‐bound vWF with rabbit anti‐vWF (DAKO) followed by Alexa 488‐ conjugated secondary antibody. The nucleus was stained with Hoechst. Samples were imaged by confocal microscopy (Leica TCS SPE) using a 40× (NA 1.15) oil objective. Maximum intensity projections were generated in Fiji and the total area of vWF staining quantified.

### 2.8 Statistical analysis

Statistical analyses were performed in GraphPad Prism version 7. Statistical significance was assessed using Student's *T* test for two sample datasets. Where more than two samples were compared, statistical significance was assessed using one‐ or two‐way analysis of variance (ANOVA) followed by Dunnet's or Sidak's multiple comparison tests, respectively. All tests were two‐tailed.

## 3 RESULTS

### 3.1 Some agonists evoke the release of large WPBs

**Figure 1:**
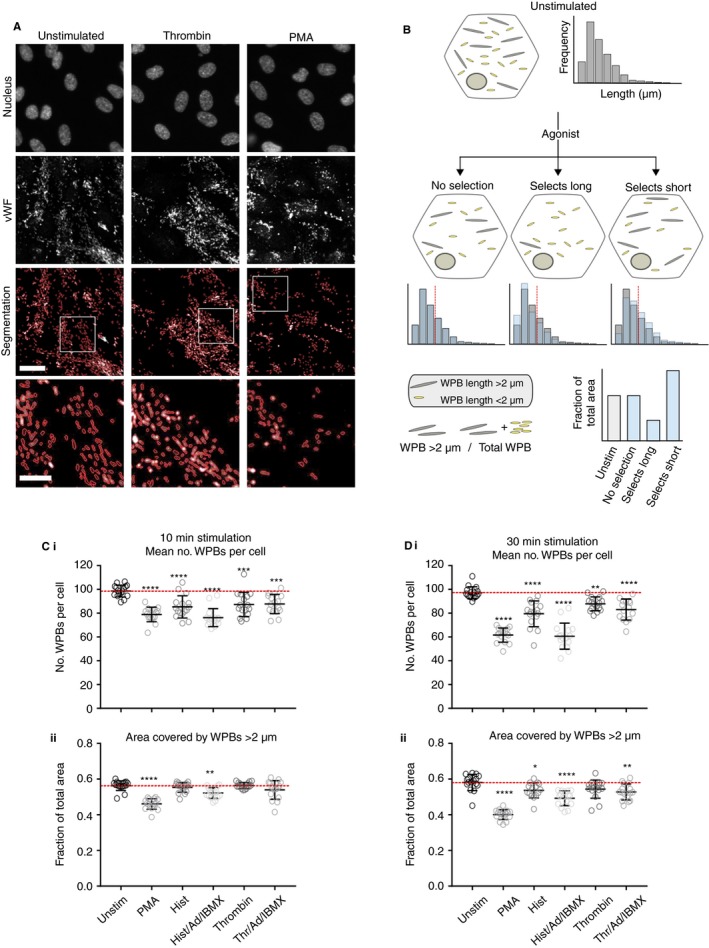
Agonists can select subpopulations of Weibel‐Palade bodies based on organelle size. A, C, and D, HUVECs were grown in 96 well plates and either unstimulated or stimulated with different agonists (PMA), Histamine (Hist), Thrombin, adrenaline (Ad), IBMX either alone or in combination as indicated, for 10 (C) or 30 (D) min before being fixed and stained for pro‐vWF and the nucleus with Hoechst (A). Up to 144 images from 16 wells were acquired per condition at 40× magnification and WPBs segmented using a custom‐designed program (Segmentation). Scale bar is 25 μm. B, Cartoon illustrating assay used to compare the effects of different agonists on WPB length distributions. Cells contain a population of WPBs of different lengths (long WPBs over 2 μm in length are in gray and WPBs shorter than 2 μm in yellow) which can be represented as in example histograms. Upon agonist stimulation WPBs will be lost from cells. If a random selection of organelles is released the length distribution will not change (left cell). The selection of longer (middle cell) or shorter (right cell) WPBs will result in the disproportionate loss of the longer or shorter WPBs. This can be seen in histograms (blue bars indicate the distributions following agonist overlaid with the example distribution from unstimulated cells). This can also be represented by looking specifically at the proportion of WPBs which are long, defined as those longer than 2 μm (dashed red line on histograms). To compare between multiple treatments the proportion of the area covered by WPBs length >2 μm is calculated as a fraction of the total area covered by all WPBs. Disproportionate loss of long WPBs will result in a fall in the area covered by WPBs over 2 μm and loss of many shorter WPBs will result in an increase in this value. Following either 10 (C) or 30 (D) min of stimulation the total number of WPBs segmented per cell (Ci, Di) and the fraction of the area covered by long WPBs (Cii, Dii) was calculated per image, and the mean of all images per well plotted (N = 16 wells). Error bars are standard error of the mean (SEM). Dotted red lines are unstimulated mean. Statistical significance was assessed with one‐way ANOVA with Dunnet's multiple comparison test. **P ≤ .01, ***P ≤ .001, ****P ≤ .0001. A representative experiment is shown from N = 4 to 7

Do endothelial agonists^2^, ^3^, ^4^ cause the release of differently sized WPBs? To test this, we measured the lengths of thousands of organelles remaining within HUVECs after activation, using an unbiased, high‐throughput imaging approach.^6^ WPBs were identified by staining for their main constituent, the processed form of vWF (pro‐vWF), and automatically segmented (Figure 1A) to search for a change in the length distribution of WPBs after exocytosis. All agonists cause a decrease in the number of WPBs per cell; if a random selection of organelles is released, the length distribution of those remaining will not differ from controls. If even some size selection occurred, we will find a differential loss of smaller, or larger, organelles (cartooned in Figure 1B). Shortening the population of WPBs to cause a loss of ~40% of WPBs longer than 2 μm is sufficient to cause a catastrophic fall in hemostatic function of the released vWF,^8^ highlighting the importance of any differential release.

Human umbilical vein endothelial cells were stimulated with a range of agonists for 10 (1C) or 30 minutes (Figure 1D). We found that by 10 minutes, both Phorbol 12‐myristate 13‐acetate (PMA), and histamine combined with adrenaline and 3‐isobutyl‐1‐methylxanthine (IBMX) consistently resulted in the differential release of long WPBs, reflected by a fall in the proportion of longer organelles remaining (Figure 1Cii). After 30 minutes, stimulation of histamine alone as well as thrombin/adrenaline/IBMX also resulted in release of long WPBs. PMA and histamine/adrenaline/IBMX each stimulated release of a similar number of organelles but markedly differed in their propensity to release long organelles, thus the loss of many organelles is not itself sufficient to drive size selection (Figure 1Dii). We also titrated the concentration of PMA, histamine/adrenaline/IBMX, and histamine and measured the number of exocytic sites (Figure S1A in supporting information) and the lengths of the WPBs remaining (Figure S1B in supporting information) and find that agonist responses are saturated at the concentrations used in Figure 1C,D and that even at higher concentrations histamine still does not produce a significant selective response following 10 minutes of stimulation.

How do different agonists produce such a selective response? WPBs are known to utilize several modes of exocytosis, including kiss and run, in which only small molecules are released (excluding vWF) and full fusion, cumulative, or compound exocytosis, in which all cargos can be released.^17^, ^18^, ^19^, ^20^ A significant proportion of WPBs undergoing full fusion also recruit an actomyosin ring, likely providing force to aid extrusion of the large (20 000+ kDa) vWF concatamers^10^, ^11^ that assemble within WPBs into multi‐concatameric assemblies that combine into strings after exocytosis. We recently found that agonists differentially recruit this ring, the most efficient of which are histamine/adrenaline/IBMX and PMA, while thrombin does not utilize an actin ring to any significant extent.^13^ We therefore hypothesized that ring recruitment may promote release of long WPBs.

### 3.2 An actin ring is required for the exocytosis of long WPBs

**Figure 2:**
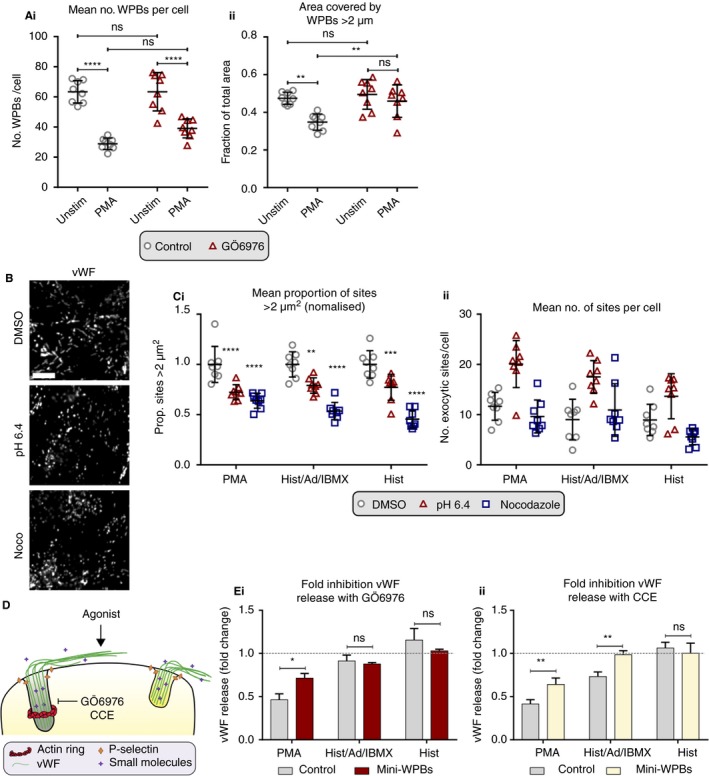
The targeting of large WPBs is dependent on recruitment of an actin ring at exocytosis. A, HUVECs were grown in 96 well plates and either untreated or treated with PKC inhibitor (GÖ6976) to inhibit formation of the actin ring. Subsequently cells were either unstimulated or stimulated with PMA for 30 min to target the release of long WPBs. Cells were fixed and stained for pro‐vWF and 72 images acquired from eight wells per condition. WPBs were segmented and the total number of WPBs segmented per cell (Ai) and the fraction of the area covered by long WPBs (length >2 μm) (Aii) calculated per image. The mean of all images per well is plotted with bars representing SEM. A representative experiment shown from N = 3 experiments. B and C, HUVECs were treated with either DMSO, or either pH 6.4 medium or nocodazole for 24 h to generate mini‐WPBs and either fixed and stained for vWF (B) or stimulated in the presence of anti‐vWF before fixation and staining for vWF (C). Images were acquired at 40× magnification, scale bar represents 10 μm. The area (Ci) or number (Cii) of exocytic sites was measured from 72 images per condition across eight wells and the average per well plotted. Ci, The proportion of sites > 2 μm^2^ was calculated and normalized to DMSO controls for each agonist. A representative experiment is shown from N = 3 experiments. Bars are SEM. D, Schematic showing the proposed use of the actin ring by long WPBs (gray). E, The amount of vWF released from control cells and cells incubated for 24 h with pH 6.4 medium to generate mini‐WPBs in the presence and absence of GÖ6976 (Ei) or CCE (Eii) was assessed by ELISA following stimulation with histamine, histamine/adrenaline/IBMX, and PMA. The amount of vWF released from cells treated with GÖ6976 or CCE is normalized to vWF release from control cells to give the fold inhibition of each treatment (1 [dashed line] indicates the inhibitor has no effect on vWF release, <1 indicates that the inhibitor has reduced vWF release compared to control untreated cells). The mean fold inhibition was calculated from N = 3 to 5 independent experiments. All bars represent SEM. Statistical significance was assessed with two‐way ANOVA with Sidak's multiple comparison test on N = 8 wells (A, C) or N = 4‐5 (Ei) and N = 3‐4 (Eii) independent experiments. *P < .05, **P ≤ .01, ***P ≤ .001, ****P ≤ .0001, ns, not significant

Is the actin ring needed for release of longer WPBs? We tested this hypothesis using the PKC inhibitor GӦ6976 as this has been previously found to perturb actin ring recruitment downstream of PMA stimulation.^13^ Incubation with the inhibitor does not significantly affect the number of WPB/cell in the absence or presence of stimulation (Figure 2Ai). However, after activation with PMA for 10 minutes, the presence of GӦ6976 prevents the release of longer WPB, because the proportion of long WPBs remaining poststimulation does not differ from unstimulated cells (Figure 2Aii). Interestingly, GӦ6976 does not block the release of long WPBs when cells are stimulated with histamine alone (Figure S2B in supporting information), or in combination with adrenaline and IBMX (Figure S2A in supporting information), suggesting that while the recruitment of an actin ring confers the ability to select long WPBs for exocytosis, that recruitment may operate via both PKC‐dependent and independent mechanisms.

### 3.3 Cells with smaller WPBs exhibit reduced actin ring usage

Our data show that first, organelle size selection does occur in endothelial cells, and is thus likely an important mechanism by which cells can modulate the hemostatic response, and second that longer WPBs may not be released if ring assembly is perturbed. WPB size is modulated in response to environmental cues via AMPK^7^ and changes in vWF expression,^8^ which is affected by vascular‐bed‐specific environmental cues.^21^ Is the ring still used if WPBs are all small? We investigated the use of the ring in cells with mini‐WPBs, generating shortened organelles by unlinking Golgi mini‐stacks via treatment with nocodazole (to depolymerize microtubules), or acetate‐containing medium at pH 6.4 (to lower the pH in the cytosol) (Figure 2B,C and Figure S3 in supporting information).^6^, ^8^ To independently confirm the relationship of WPB size and ring usage, we used an assay developed to measure the area of WPB exocytic events,^13^, ^15^ which shows larger exocytic sites correlating with actin ring use.^13^ This confirmed that both shortening treatments reduced the number of large exocytic sites in response to all agonists (Figure 2Ci). Interestingly, this is coincident with pH 6.4 treatment increasing overall exocytic activity (Figure 2Cii).^8^

We also tested the effect of short WPBs on vWF secretion. We noted that GӦ6976 and CCE (which is less selective but also inhibits ring formation) both inhibit the release of vWF in PMA‐stimulated cells.^10^, ^13^ If this inhibition reflects loss of actin ring function, and the ring is indeed required for the release of long WPBs, then exocytosis in cells with shorter WPBs should be less affected by these treatments (Figure 2D). Cells grown for 24 hours in medium at pH 6.4 containing acetate to generate “mini‐WPBs” (the proportion of vWF area covered by long WPBs [length > 2 μm] was reduced by approximately half), were stimulated with different agonists in the presence or absence of GӦ6976 (Figure 2Ei and Figure S4A in supporting information) or CCE (Figure 2Eii and Figure S4B in supporting information) and the amount of vWF released measured. We calculated the efficacy of the two inhibitors by dividing the amount of vWF released from inhibitor‐treated cells by the amount released in the corresponding untreated cells to give a fold inhibition (a value of <1 indicates less vWF was released from cells treated with inhibitor than controls, a value of 1 indicates the inhibitor had no effect on release, while >1 indicates that more was released from cells incubated with inhibitor than control cells).

**Figure 3:**
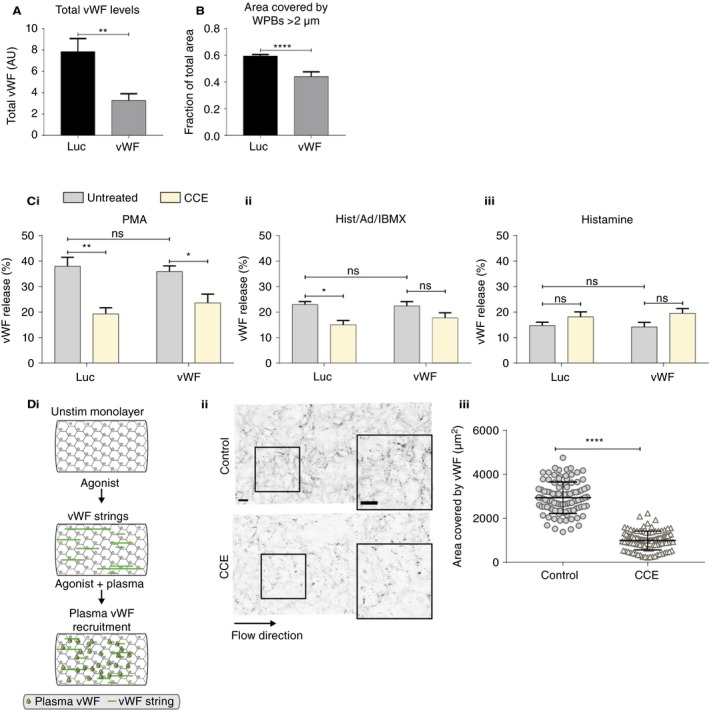
Modulating the WPB population alters exocytic mode. HUVECs were nucleofected with siRNA against Luciferase (Luc) or vWF. A, The total amount of vWF was measured by ELISA from N = 12 independent experiments (bars are SEM). B, The mean area covered by WPBs with length >2 μm was calculated. A representative experiment is shown from N = 6 independent experiments (bars are SD). C, The mean percentage of vWF released from Luciferase and vWF‐depleted cells in the presence and absence of CCE was assessed by ELISA following stimulation with PMA (Ci), histamine/adrenaline/IBMX (Cii), and histamine (Ciii) (from N = 4 independent experiments). Bars represent SEM. D, HUVEC monolayers under flow were stimulated with histamine in combination with adrenaline and IBMX in the presence of human pooled plasma and in the presence or absence of CCE. Di, Schematic of flow assay workflow. Dii, Cells fixed and stained for external vWF. Images were acquired at 40× magnification. Scale bars represents 50 μm. Diii, Quantification of the area covered by vWF per image (N = 50‐100 images per experiment) and a representative experiment from N = 3 independent experiments is shown. Statistical significance was assessed with Student's *T* test (A‐B, D) or two‐way ANOVA with Sidak's multiple comparison test (C). *P < .05, **P ≤ .01, ****P ≤ .0001, ns, not significant

Results confirmed the prediction that the degree to which GӦ6976 and CCE inhibit release in control cells strongly correlates with actin ring recruitment and a propensity to release long WPBs. PMA, the agonist most associated with exocytosis of long WPBs and with ring recruitment, is most inhibited by both drugs; vWF release falling by approximately 50% in control cells (Figure 2Ei,ii, gray bars and Figure S4), whereas histamine, the least associated with exocytosis of long WPBs, is not inhibited by either drug. Histamine/adrenaline/IBMX, in line with previous data (Figure S2A), is not inhibited by GӦ6976, but release is reduced to about 30% of control cells upon CCE treatment. This is likely because histamine activation may not require PKC.^22^ We thus find that in cells with mini‐WPBs, blocking the actions of the actin ring has a minimal effect on vWF release. Histamine/adrenaline/IBMX‐stimulated cells with mini‐WPBs are no longer inhibited by CCE treatment, while PMA‐stimulated cells with mini‐WPBs are significantly less affected by either inhibitor treatments than control cells (vWF release is inhibited 50% in control cells and only ~25% in cells with mini‐WPBs for both drugs) (Figure 2E). Indeed, in all cases, cells with mini‐WPBs show no significant change in the amount of vWF released compared to control untreated cells (Figure S4). These data are entirely consistent with our conclusion that the actin ring is specifically required for the release of long WPBs.

von Willebrand factor expression varies between vascular locations in vivo.^23^ To model the effects of changes in vWF levels we titrated vWF siRNA to give a 60% depletion in vWF protein levels (Figure 3A), reducing the fraction of long WPBs by 25% (Figure 3B).^8^ We then measured the amount of vWF released following stimulation with PMA, histamine/adrenaline/IBMX, and histamine in the presence or absence of CCE (Figure 3C). No change was seen in the total amount of vWF released following agonist stimulation in control cells suggesting that while there is a bias towards= long WPBs being released following PMA and histamine/adrenaline/IBMX stimulation, if these are not available shorter WPBs will be released. Again, we find that even a small change in WPB size makes PMA and histamine/adrenaline/IBMX‐stimulated cells less sensitive to CCE treatment, further indicating the use of the actin ring is most necessary for release of long WPBs.

### 3.4 Failure to release long WPBs inhibits haemostatic functioning

What are the functional consequences of reducing the release of the longest WPBs? We have previously shown that cells with small WPBs produce disproportionately fewer and shorter strings and recruit less plasma vWF and platelets (both necessary for hemostasis).^6^, ^8^ We therefore hypothesized that inhibiting the release of long WPBs, which numerically make up only a small proportion of exocytic events following stimulation, would also have a disproportionate effect on function. We previously found that using an acute low dose of CCE to inhibit actin ring formation in activated cells under flow results in the production of significantly shorter vWF strings.^13^ We now stimulated cells with histamine/adrenaline/IBMX under flow in the presence or absence of CCE and assessed the ability of agonist‐activated cells to recruit plasma vWF (Figure 3Di). A highly significant reduction in the recruitment of plasma vWF occurs when CCE is used to inhibit the actin ring to inhibit the release of long WPBs (3D). Thus, loss of the differentially increased release of longer WPBs at exocytosis by some agonists, even to a relatively modest extent (histamine/adrenaline/IBMX results in around a ~15% fall in long WPBs compared to unstimulated cells [Figure 1Dii]) has a significant impact on hemostatic function: a 60% fall in the recruitment of plasma VWF to cells.

## 4 DISCUSSION

**Figure 4:**
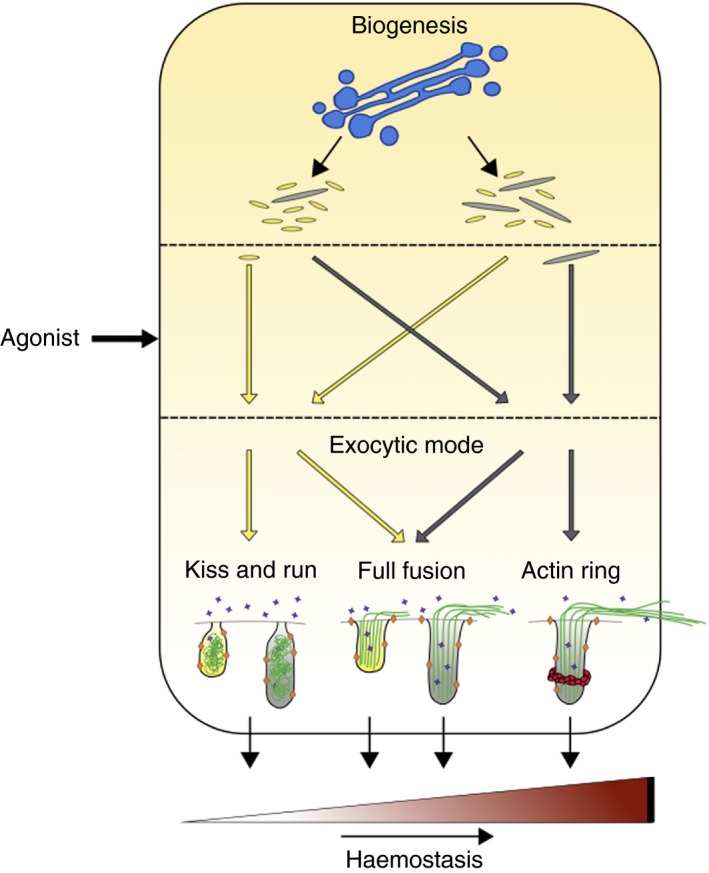
Internal and external control points that influence endothelial output. Schematic summarizing the proposed control points that can influence the magnitude of the hemostatic response. Endothelial cells can drive the biogenesis of shorter (yellow) or larger (gray) organelles at the Golgi (blue), therefore controlling the availability of longer prothrombotic WPBs. External stimuli influence the response via selection of organelles based on size. Finally, the exocytic machinery selected controls the amount of vWF released. Kiss and run exocytosis results in the release of small molecules (purple stars) with vWF (green) failing to be exocytosed. This mode of exocytosis can presumably occur from WPBs of all lengths (left). Full fusion of WPBs releases small molecules, membrane proteins (orange diamonds), and vWF, but strings produced from cells with populations of shorter WPBs are fewer and shorter, while the strings produced from cells in which the actin ring is inhibited are shorter (center). The recruitment of an actin ring to the largest WPBs confers the most prothrombotic response (right)

This investigation into exocytosis of WPBs in primary human endothelial cells reveals that some agonists evoke the selective release of content from larger WPBs (summarized in Table S1 in supporting information), providing a novel layer of secretory control. Automated high‐throughput imaging of thousands of organelles (>150 000 per condition; distributed between 16 units of analysis) combined with biochemical measurements of endothelial secretion shows that WPB size influences both likelihood and mode of exocytosis in response to different agonists. We have also recently demonstrated cellular control of WPB size in response to environmental cues, coordinated by AMPK^20^: coupling cellular machineries that control organelle size to those supporting a range of exocytic modes allows for highly differentiated functional responses to be generated by endothelial cells from the release of a single type of organelle (Figure 4).

Endothelial agonists can broadly be divided into two classes, those that act by raising intracellular levels of calcium and those using cyclic AMP (cAMP). However, how approximately 30 different agonists can facilitate different responses following endothelial activation is not clear. While it has been proposed that cAMP‐agonists, by only targeting peripheral organelles,^24^ can provide differential responses, it is unlikely that this would provide such an output because all WPBs are packed with similar sets of cargo (noting that the content of newly forming WPBs in any cell will be simultaneously affected by changes in expression of certain cargos, eg, IL‐1β triggering upregulation of IL‐8^25^, ^26^ or Interleukin‐4 and oncostatin M upregulating P‐selectin.^27^ Our new findings show how a range of agonists can indeed provide differentiated responses but through exploiting the functional differentiation generated by a range of granule sizes, rather than as previously suggested, by different contents for individual WPBs.^28^

Several cell types produce secretory granules of different sizes—albeit none on anything like the ten‐fold scale of difference seen in the endothelium. These include the dense‐core granules of neuroendocrine cells, and secretory granules of mast and pancreatic acinar cells, among others.^29^, ^30^, ^31^, ^32^ It is therefore possible that other secretory cells also exploit size differences to modulate output. While difficulty in measuring the size of spherical organelles makes investigation of this question technically challenging in most other cell types, a link between granule size and exocytic mode has been demonstrated in PC12 neuroendocrine cells. Here, isoforms of the calcium sensor synaptotagmin localize to differently sized granules which in turn use different modes of exocytosis.^33^ Further, a preference for full fusion over kiss and run was also seen in larger vesicles from lactotrophs stimulated with sphingosine.^34^ Our data, from a much more explicitly size‐driven system, complement these examples and suggest that using granule size to regulate secretory function may be widespread. However, because for most cargos the functional effect of size selection will only be proportional to the amount released, and the range of sizes is much smaller, the functional consequences of this phenomenon may not be so significant. Further, size selection can only arise where a fraction of granules is used at activation; where all organelles are simultaneously used (such as in extreme compound exocytosis^35^), this strategy would be moot.

How granule size is sensed for selection is unclear. Studies have demonstrated that differential sensitivity to calcium can be used to release distinct subpopulations of granules.^36^ This, in principle, could operate by differential localization of fusion machinery to subgroups of granules. However, while differential localization of SNAREs^37^ potentially provides a mechanism for differential exocytosis, this does not solve the problem of how SNAREs are differentially delivered to organelles distinguished only by their size. While WPB length is highly variable, the width of these organelles is remarkably uniform; thus, features such as membrane curvature are unlikely to act as differentiators between differently sized WPBs. Furthermore, WPBs fuse with the plasma membrane at their tips, which would also preclude the possibility that an increased number of SNARE molecules are present on longer organelles.

In vivo the likelihood that endothelial activation results from a single agonist is highly unlikely. For example, adrenaline is always present in plasma.^38^ Thus, our findings of differences in vWF release between single and combined agonists are likely important in understanding how endothelial activation occurs in a physiological setting. While PMA itself is not a physiological agent, the similar results we find with histamine/adrenaline/IBMX suggest that PMA can still be a useful tool to identify underlying mechanisms. Although it has been shown that agonists may act synergistically to promote the release of vWF,^13^, ^39^ most work has examined the effects of a few agonists in isolation, such that downstream signaling pathways activated by multiple agonists are largely unexplored. Given that in vivo, endothelial activation resulting from a single agonist is unlikely, this is surprising. Here, we show that addition of adrenaline and IBMX (which stimulate cAMP production) to either histamine or thrombin (calcium‐raising agents) promotes release of longer WPBs above either alone. Variations in the levels of agonists such as raised adrenaline in a “fight or flight” response could thus amplify an individual's response to an injury causing endothelial activation. Conversely, inhibiting recruitment of the actin ring should specifically inhibit the release of the most thrombotic subset of organelles: patients undergoing vascular surgery, or suffering from other thrombosis‐related disorders might benefit from prophylactic use of such therapies. In another physiological context, vWF plays a role in cancer metastasis^40^, ^41^, ^42^ in which increased levels of vWF are associated with poor prognoses in acute myeloid leukaemia and distant metastases in lung cancer patients.^43^, ^44^ Altogether, maximum flexibility in regulating vWF release could prove therapeutically beneficial in numerous conditions.

In summary, we have identified a new layer of regulation that may allow endothelial cells to maximize the range of functional outputs that can occur following activation. The resulting highly tunable system can not only respond to a variety of cues, but also offers several checkpoints that could potentially reduce the likelihood of aberrant activation occurring. We have outlined a system in which granule size, exocytic mode, and agonist‐specific targeting have been shown to be coupled together to influence measured functional output. WPB size is becoming increasingly apparent as a key player in modulating endothelial output and this work provides another part of the mechanism by which this arises.

## CONFLICT OF INTEREST

All three authors were funded by an MRC grant MC_UU_12018/2 awarded to DF Cutler. All three authors state that they have no conflicts of interest.

## AUTHOR CONTRIBUTIONS

Jessica J. McCormack: conceptualization, data analysis, investigation, methodology, writing original draft, reviewing, and editing. Kimberly Harrison‐Lavoie: investigation, data analysis. Daniel F. Cutler: conceptualization, Funding acquisition, project administration, supervision, writing original draft, reviewing, and editing.
