# Supplementary material for: Human endothelial cells size‐select their secretory granules for exocytosis to modulate their functional output
Source: J Thromb Haemost. 2019 Oct 2;18(1):243–54. doi: 10.1111/jth.14634 (PMC7155122; doi:10.1111/jth.14634)
Supplement: Supplementary file 1 [file JTH-18-243-s001.pdf]

Supplementary Figure 1

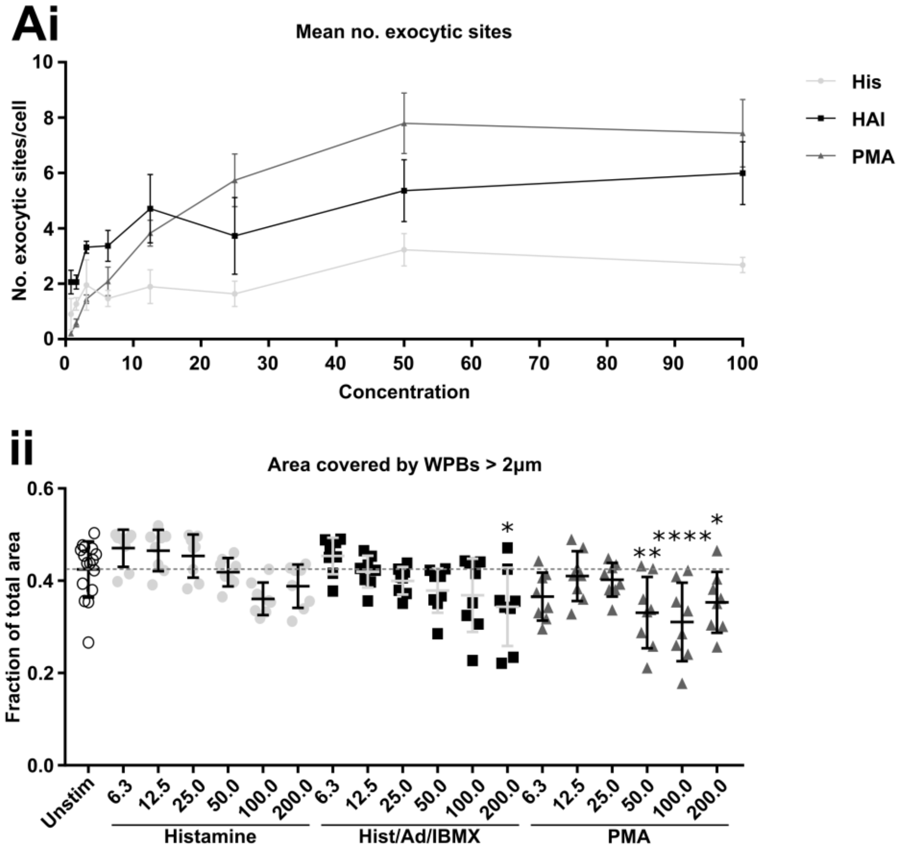

Supplementary Figure 1:

HUVECs were grown in 96 well plates and stimulated with increasing concentrations of agonists (units are µM for histamine and histamine/adrenaline/IBMX and in ng/ml for PMA) as indicated in either the presence of anti-vWF to visualise exocytic events (Ai) or fixed and subsequently stained for pro-vWF to visualise WPBs remaining in cells (Aii). Ai) The number of exocytic events per image was quantified and the mean number of sites per cell per well plotted with bars representing SEM (N=8 wells, a representative experiment shown from N=3 independent experiments). Aii) The fraction of the area covered by long WPBs (length greater than 2µm) was calculated per image and the mean fraction of the area per well plotted from N=8 wells with bars representing SEM. A representative experiment is shown from N=3 experiments. Statistical significance was assessed using ANOVA with Dunnet's multiple comparison test. * $P<0.05$, ** $P \leq 0.01$, **** $P \leq 0.0001$.

Supplementary Figure 2

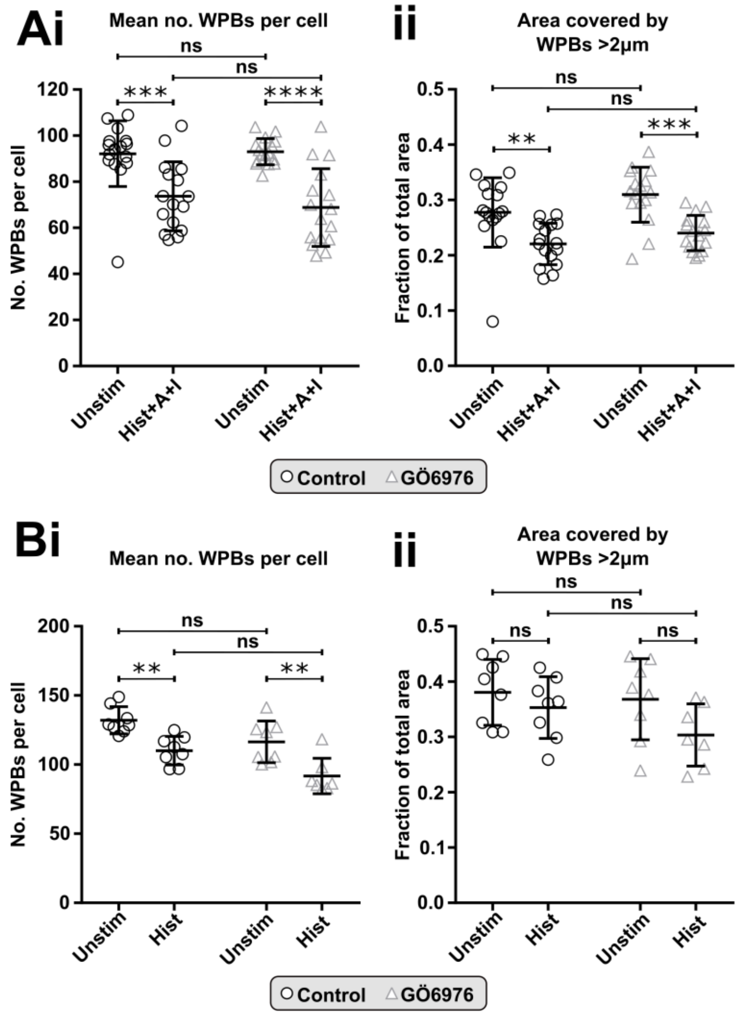

Supplementary Figure 2:

HUVECs were grown in 96 well plates and either untreated or treated with the PKC inhibitor GÖ6976 to inhibit formation of the actin ring. Subsequently cells were either unstimulated or stimulated with histamine/adrenaline/IBMX (A) or histamine alone (B) for 30 minutes. Cells were fixed and stained for vWF and 72 images acquired from 8 wells per condition. WPBs were segmented and the total number of WPBs segmented per cell (Ai, Bi) and the fraction of the area covered by long WPBs (length greater than 2μm) (Aii, Bii) calculated per image. The mean of all images per well is plotted with bars representing SEM. A representative experiment shown from N=3-4 experiments. Statistical significance was assessed with two-way ANOVA with Sidak's multiple comparison test on N=8 wells (Ai-ii, Bi-ii). ** P≤0.01, *** P≤0.001, **** P≤0.0001, ns = not significant.

Supplementary Figure 3

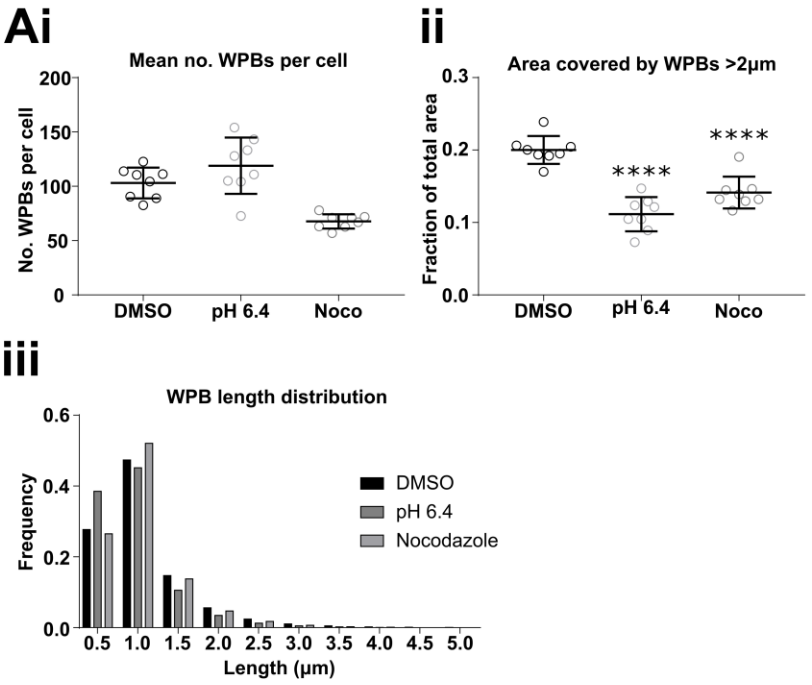

Supplementary Figure 3:

HUVECs grown in 96 well plates were incubated with either DMSO, nocodazole (Noco) or pH 6.4 media overnight. Cells were fixed and stained for pro-vWF and 72 images acquired across 8 wells per condition. WPBs were segmented and the mean number of WPBs per cell (Ai) and area covered by long WPBs (length >2µm) (Aii) plotted per well. The length distributions were also plotted as a histogram (binning at 0.5µm) (Aiii). Statistical significance was assessed with one-way ANOVA with Dunnet's multiple comparison test. **** P≤0.0001.

Supplementary Figure 4

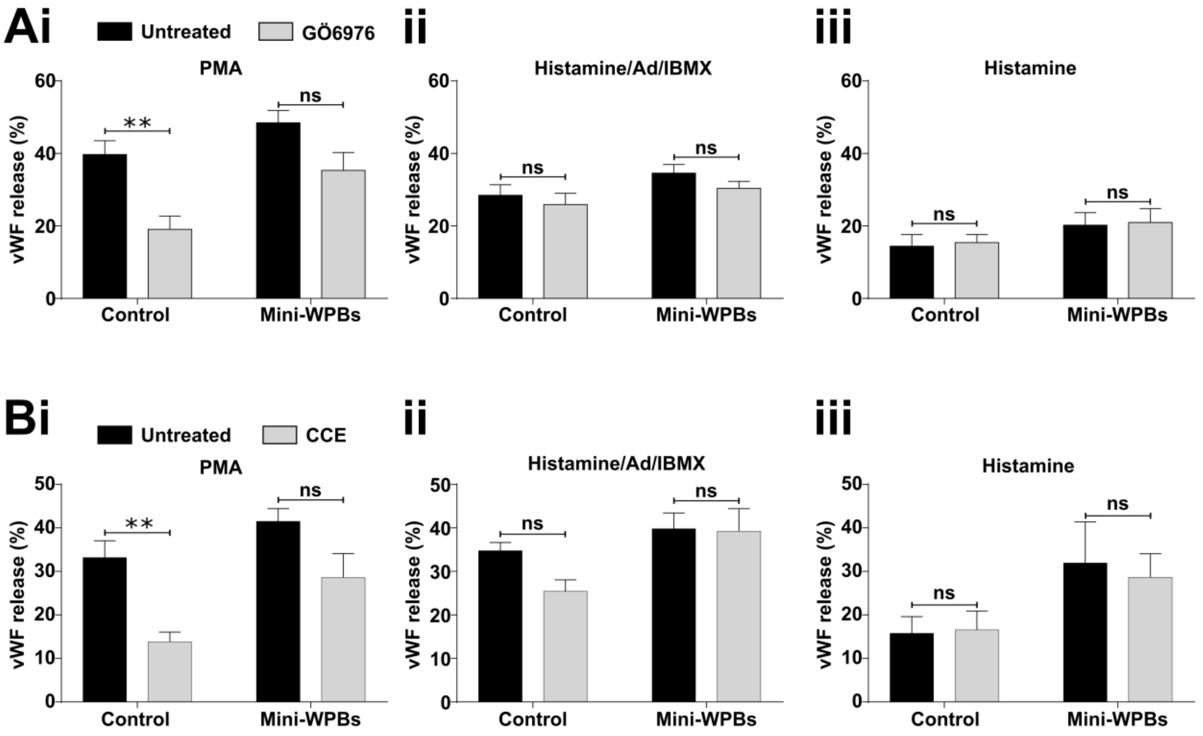

Supplementary Figure 4:

The amount of vWF released from control cells and cells incubated for 24 hours with pH 6.4 medium to generate mini-WPBs in the presence and absence of GÖ6976 (A) or CCE (B) was assessed by ELISA following stimulation with histamine, histamine in combination with adrenaline and IBMX and PMA. The mean amount of vWF released from n=3-4 independent experiments is shown. Normalised data are shown in Figure 2C-D. Statistical significance was assessed with two-way ANOVA with Sidak's multiple comparison test. ** P≤0.01, ns = not significant.

Supplementary table 1: Summary of agonist effects on endothelium

Agonist	Induces release of long WPBs?	vWF release inhibited by blocking actin ring	Functional consequences
Histamine	Yes*	No	
Histamine/Adrenaline/IBMX	Yes	Yes	Reduction in plasma vWF recruitment
Thrombin	No		
Thrombin/Adrenaline/IBMX	Yes		
PMA	Yes	Yes	

*Very minimally
